# Social Determination of the Health of Families from Two Neighborhoods in Medellín, Colombia* ** ***

**DOI:** 10.17533/udea.iee.v40n2e14

**Published:** 2022-09-20

**Authors:** Dora Lucía Gaviria Noreña, Rusbert Fernando Álvarez Del Río, Nora Eugenia Zapata Gómez, Maribel González Giraldo

**Affiliations:** 1 Nurse, Master’s. Professor. Faculty of Nursing, Universidad de Antioquia, Medellín (Colombia). Email: dora.gaviria@udea.edu.co Universidad de Antioquia Faculty of Nursing Universidad de Antioquia Medellín Colombia dora.gaviria@udea.edu.co; 2 Nurse, Master’s. Professor. Faculty of Nursing, Universidad de Antioquia, Medellín (Colombia). Email: rusbert.alvarez@udea.edu.co Universidad de Antioquia Faculty of Nursing Universidad de Antioquia Medellín Colombia rusbert.alvarez@udea.edu.co; 3 Nurse, Master’s. Professor. Faculty of Nursing, Universidad de Antioquia, Medellín (Colombia). Email: nora.zapata@udea.edu.co Universidad de Antioquia Faculty of Nursing Universidad de Antioquia Medellín Colombia nora.zapata@udea.edu.co; 4 Nursing Student. Email: maribel.gonzalezg@udea.edu.co Faculty of Nursing, Universidad de Antioquia, Medellín (Colombia). Universidad de Antioquia Faculty of Nursing Universidad de Antioquia Medellín Colombia maribel.gonzalezg@udea.edu.co

**Keywords:** community health nursing, social determination of health, health-disease process, public health, human migration, enfermería en salud comunitaria, determinación social de la salud, proceso salud-enfermedad, salud pública, migración humana., enfermagem em saúde comunitária, determinação social da saúde, processo saúde-doença, saúde pública, migração humana.

## Abstract

**Objective.:**

To understand the health process, from the approach of the social determination of health in two neighborhoods in Medellín - Colombia, to contribute to the care of people, families, and collectives in their multidimensional reality.

**Methods.:**

Qualitative research from the ethnographic perspective, approaching the general dimension with documentary analysis of social policies and community documents, the particular dimension through focal groups and interviews to community leaders, and the singular dimension with the family visit.

**Results.:**

Families and collectives live within a sociocultural setting of resistance, overshadowed by moments of flight and displacement derived from violence, with scant participation in city plans and programs and with structural problems of economic and political exclusion. They constructed the territory as space and shelter in the weave that protects and violates them, with processes from uprooting to rooting. The families have maintained protective processes, like family participation in decision making, knowledge on health care, among others, and destructive processes, like informal labor and job instability, without spaces for recreation and with limitations in transportation, in access to health programs and in obtaining food.

**Conclusion.:**

The health of the families has been determined by historical exclusion to work to obtain resources for a minimum vital subsistence, which is why they suffer social vulnerability due to few opportunities for development; they have lived a transformation process of the territory with resistance, solidarity, and construction of social networks.

## Resumen

**Objetivo.:**

Comprender el proceso salud, desde el enfoque de la determinación social de la salud en dos barrios de Medellín, para contribuir al cuidado de las personas, familias y colectivos en su realidad multidimensional.

**Métodos.:**

Investigación cualitativa desde la perspectiva etnográfica; abordó la dimensión general con análisis documental de políticas sociales y documentos comunitarios, la dimensión particular a través de grupos focales y entrevistas a líderes comunitarios y la dimensión singular con la visita familiar.

**Resultados.:**

Las familias y los colectivos viven en un espacio sociocultural de resistencia, matizados por momentos de huida y desplazamiento derivados de la violencia, con escasa participación en los planes y programas de ciudad y con problemas estructurales de exclusión económica y política. Construyeron el territorio como espacio y refugio en la urdimbre que los protege y vulnera, con procesos del desarraigo al arraigo. Las familias han mantenido procesos protectores como la participación familiar en toma de decisiones, conocimientos del cuidado de la salud, entre otros, y procesos destructivos como el trabajo informal e inestabilidad del empleo, sin espacios para la recreación y con limitaciones en el transporte, en el acceso a programas de salud y en la consecución de alimentos.

**Conclusión.:**

La salud de las familias ha estado determinada por la exclusión histórica al trabajo para obtener los recursos para un mínimo vital de subsistencia, por lo que sufren vulneración social por escasas oportunidades para el desarrollo; han vivido un proceso de transformación del territorio con resistencia, solidaridad y construcción de redes sociales.

## Resumo

**Objetivo.:**

Compreender o processo de saúde, a partir da abordagem da determinação social da saúde em dois bairros de Medellín, para contribuir com o cuidado de indivíduos, famílias e grupos em sua realidade multidimensional.

**Métodos.:**

Pesquisa qualitativa na perspectiva etnográfica; abordou a dimensão geral com análise documental de políticas sociais e documentos comunitários, a dimensão particular por meio de grupos focais e entrevistas com lideranças comunitárias e a dimensão singular com a visita familiar.

**Resultados.:**

Famílias e grupos vivem em um espaço sociocultural de resistência, matizado por momentos de fuga e deslocamento derivados da violência, com pouca participação nos planos e programas da cidade e com problemas estruturais de exclusão econômica e política. Construíram o território como espaço e refúgio na urdidura que os protege e os viola, com processos de desenraizamento ao enraizamento. As famílias têm mantido processos protetivos como a participação da família na tomada de decisões, o conhecimento dos cuidados de saúde, entre outros, e processos destrutivos como o trabalho informal e a precarização do emprego, sem espaços de lazer e com limitações no transporte, no acesso aos programas de saúde e na obtenção de alimentos.

**Conclusão.:**

A saúde das famílias tem sido determinada pela exclusão histórica do trabalho para obtenção de recursos para um mínimo vital de subsistência, pelo qual sofrem vulnerabilidade social devido às escassas oportunidades de desenvolvimento; vivenciaram um processo de transformação do território com resistência, solidariedade e construção de redes sociais.

## Introduction

Achieving the well-being of humans has been the interest of health sciences, specially of nursing, which has advanced in comprehending the health-disease process^1^ through theoretical, investigative, and practical construction, in search of caring for people, families, and communities, in complex sociocultural settings woven into everyday life.^2^

This research emerged from two neighborhoods of the 15 that make up commune three of the city of Medellín, Colombia, on the formation of nursing students and with the construction of solidarity projects at Universidad de Antioquia. The work assumed the approach of the social determination of health from the critical epidemiology to comprehend the health process in its complexity configured from three dimensions or domains: general, particular, and singular,^3^,^4^ and which establishes the ways economic, social, and political relations are produced and reproduced. Eslava^5^ states that social determination is a holistic and dynamic view of the social events that affect life. 

The people, families, and communities that inhabit the territory of the neighborhoods La Honda and La Cruz, peripheral zone of the city, have experienced historical and political processes that have conditioned in differential manner their ways of living, getting sick, and dying for over three decades and after suffering displacement, as stated in their community diagnosis by the Community Organizations Network of the La Cruz and La Honda neighborhoods (RIOCBACH, for the term in Spanish);^6^ hence, requiring to document and recognize these processes, seeking to provide health care actions. For the families, the scourge of fleeing their homelands due to acts of violence has marked significantly their life trajectories, reconfiguring new settlements in the city slopes, under unfavorable conditions for their well-being, dignity, inclusion, and democracy^.(^6

Knowing the determination of the health of these families permits understanding the current conditions and urban-popular population dynamics that affect ownership and the political-administrative recognition as neighborhoods, access to health and social services, quality of housing, education, work, and possibilities for personal and collective development, as evidenced by other studies that have shown situations of inequality of the people who live in peripheral zones, affected by phenomena of the armed conflict, displacement, and the devastating land dispossession, which even has, throughout the country, a historical accumulation of nearly 8.1 million victims from 1985 to 2020.^7^ Some investigations on the social determination of health reveal the problems of urban and rural communities in Colombia, as is the case of Indigenous peoples, according to Lozano and Salazar^8^ and Ramírez *et al*.;^9^ malnutrition and impaired child development due to historical poverty, in works by Carmona;^10^ and the worsening of the health situation of the child population with disabilities, exposed by Hurtado and Arrivillaga.^11^

In this sense, the social determination of health provides knowledge, methods, and techniques for critical reflection, contextual and complex multidimensional analysis of health processes, caring for life, well-being, and collective health; all crucial for the effectiveness of policies, programs, and projects that seek to promote health and prevent disease in human groups. The aim of this research was to understand the health process of families, from the approach of social determination; focusing its interest on dynamics of adaptation and solidarity weaved and which permit survival, to contribute to the comprehensive care of people and collectives in their multidimensional reality; besides contributing to the planning and management of territorial health. 

## Methods

Qualitative research from the ethnographic perspective; data collection and analysis integrated different techniques and instruments that permitted the complex and holistic comprehension of the social determination of the health of families from two neighborhoods in Medellín from 2019 to 2020. The general dimension contemplates the historical logics of political, economic, ideological, and cultural order that comprise the social structure.^3^,^12^ Documentary analysis was performed on 55 planes, policies, programs or projects, memories, diaries, cartographic material and other documents, and complemented with interviews, which accounted for how the country, the department of Antioquia, and the city of Medellín have been affected with the phenomena of violence and displacement.

Interpretation of this dimension was conducted through Walt’s analytical proposal^13^ which approaches the analysis of public policy and which is pertinent to reveal the relations and dynamics present in the general dimension; the author proposes five analysis categories: context, process, content, actors, and effects. A matrix was constructed that permitted the organization, classification, comparability, and analysis of content and identified emerging categories and subcategories. The particular dimension comprises the relational processes of the social groups, which take place due to the structural logics and explain the possibilities of their ways of life, which according to Breilh^3^ and reaffirmed by Carmona^14^^) “^establishes the mediation conducted by the groups in their creative and resilient action to progress in the conquest of their rights; social class, gender, and ethnicity, are determinant in the ways of life”. This dimension was identified through the focal-group technique, aimed at identifying needs, practices, and actions carried out by social and community groups that inhabit the territory in the daily transformation they confront to solve their collective problems.

Three focal groups were held with participation by seven leaders from the network of caregivers and two interviews to members of the Community Action Board to understand their ways of life; this was complemented with a reflexive process with observation and field diaries. The information was coded and interpreted in search of meanings to, then, construct the subcategories and recognize the central category; also creating the analytical memos and conceptual maps. The singular dimension expresses that which “corresponds to the individual, where the genotype and phenotype are located in binding manner with the general and particular levels, determining the lifestyles”^15^ This was conducted through the observation and family visit that adopted and updated the instrument of the Primary Health Care model of Antioquia.^16^

The families were selected through invitation, in a non-probabilistic sample; choosing 40 families with which there were links through the community leaders and support networks, or whose situation of vulnerability required their prioritization for social and health care. The information was grouped into the following categories: sociodemographic aspects, housing characteristics, and protective and unhealthy processes**.** The analysis was performed through descriptive statistics, using Office Excel and SPSS version 25.

The research was of minimum risk and had a process of ethical reflection, respecting the right to freely participate in the study, as well as dignified and comprehensive treatment of the participants. Authorization and signed informed consent were obtained from the members of the Community Action Board, the health care givers network, and the participating families. 

A reflexive meeting was carried out with the community and the booklet “I take care of myself, we take care of ourselves and we take care of our territory” was delivered as contribution to the social appropriation and to the gratitude with the territory. The research was approved by the Research Ethics Committee and the Technical Research Committee of the Faculty of Nursing at Universidad de Antioquia.

## Results

### General dimension: political, social, and economic actions and their expression in the territory

The general dimension analyzed the historically imbricated reality through political, social, and economic actions that have transformed cultural practices in the constitution of the territory of both neighborhoods, as places and spaces for the reception of displaced families. The people and families who live there had their lands taken from them; their experiences for over 20 years have been overshadowed by moments of flight and displacement from the departments of Chocó, Córdoba, and Antioquia; they have come to take refuge on the slopes of the city, which has allowed them to create a diverse socio-cultural space. In the new neighborhoods they inhabit, they have been witnesses and builders of the physical, social, and cultural transformation, with resistance and solidarity processes that today leave a mark in the city, with cultural practices that have saved their community from disappearance, eviction, relocation, and concealment. In the participatory diagnosis document, community leader Cárdenas Avendaño expresses:^17^ *I arrived 20 years ago to the neighborhood, to the hillside, it took me in with my family, it was the shelter against our flight. To this live space I owe my experience and my struggles. It was in the neighborhood where I learned of the invitation to work, solidarity, of giving and receiving, of persistence; women and men that have been maintained, remained and resisted as the mountain does with the neighborhood, that mountain that shelters us.*

In the absence of public policies, inclusive and continuous city plans and programs in the territory, community mobilization has generated territorial autonomy through collective actions, like invitations and communal work; they have had achievements, such as access to public transportation, drinking water, inviting people from both neighborhoods to work in the physical infrastructure and sanitation works, construction of the school, house of prayer, and main road; work is still under way to complete the construction of the sewer system, waste disposal and garbage collection, access roadways, sports venues, health services, and property legalization.

Even now, their space unveils historical inequalities, compared with other communes in Medellín, like El Poblado, where the Multidimensional Index of Living Conditions (IMCV, for the term in Spanish) is 76.6 and that of commune #3, Manrique, is 37.5;^18^ which hinders the survival of over 20,000 who live there, most with wages from informal labor, routes to the city center, and some from drug micro-trafficking, which although occult in the territory, continues being one of the ways that most seduces mainly the youth in search of their economic support.

The process of implementing and contents of policies, plans, and programs in the territory has been fragmented and not prioritized; although at the beginning of the constitution of the geographic space, the participation process was strong, as expressed by the leaders, at the moment it vanishes due to the lack of new leadership, wear of the first inhabitants and, in the technical, due to the political-administrative division of the city to assign resources granted for the entire commune, with less possibilities for the neighborhoods of La Cruz and La Honda; with a trend during the last decade toward incorporating technology, but still with limitations in access for people from the outskirts.

Analysis of the interactions by the players in city programs and projects that include these neighborhoods indicated those who formulate policies, who are usually part of political-governmental organizations; who execute programs and projects, like interdisciplinary work teams; and, finally, converge people and community leaders, who receive the effects of the policies, with scarce opportunity in decision-making settings. Comprehension of the general dimension and its articulation with the particular and singular was expressed by the persistence of critical processes derived from displacement due to violence against people, families and territory collectives. This is the most dramatic experience they have endured and this condition has worsened their economic situation, which has brought them unemployment, hunger, and poverty; they have had to build again with minimal resources available to them.

### Particular dimension: the weave between that which protects us and makes us vulnerable

This dimension contemplated the ways of life of the work of groups of women and men who have woven with their hands the space, as shelter for their survival; the central category *from uprooting to rooting* emerged from the focal groups, which unveiled voices, creations, expressions they experience daily and give sense and meaning to the collective and reflecting an imminent need for the other. In the words by one of the participants of the focal group: *I came to a neighborhood that is also of much violence, of hit men and gangs, that is, the people here in Medellín we all come from a municipality, from towns, from farms, right? Here, we got somewhat civilized because we study, work, we relate because there are more ways to do so.* Another participant expressed: *I am from a town in Antioquia; I am displaced from Urabá, I lived there for 25 years; because of the violence, we had to come here to Medellín; displacement is permanent in our communities, families constantly arrive here at the hill with nothing.*

The path to rebuilding and returning to being has been conceived a trajectory of suffering upon the death of a loved one, of the dispossession of their land, the roofs over their heads, their sustenance, their social groups, their work, of belonging, of leisure, and of freedom; as one of the participants stated: *It is like being in a hole*, experience that prompted them to transform, with the stamp of longing, of common stories and of the collective mourning that is still present, even if the situation was experienced two or 20 years ago. 

The transformation takes place fundamentally from the being and the new territory allows them to construct a better future; they are peasant families that return to plow the mountain, with a leading role by women, who have been and continue being support in life processes and practices. They have assumed care and the responsibility of protection: they feed, struggle, lead, build homes, paths and through invitation reconstruct the territory in a before and after. As stated by one of the female leaders: *it was very hard for us to enter those work sites so that the car could build the roads, because that was like a bridle path; we cleared that with a pick and shovel, we used to get together up to 150 people to work.*

Rooting and uprooting are two interrelated concepts that have been woven in everyday life, with intercultural processes; they have been forged in solidarity, union and in generating ties and bonds; as expressed by one of the participants: *we got here in different ways, some with nothing and others with almost nothing, but we are all one, to subsist and take over the hill.* They created the territory with their own hands and, although they are fine because they so state it, they continue to evoke moments lived, places, people, paths and their lands. The dynamics that have been constructed in the new territory determine living in community, which favors permanence and salvation in search of shelter to face their dispossession that left them without any material possession, with fear and aimlessly; now there are new roads, houses, schools, collective spaces. In collective work, they have constructed shelters for their families; materials, such as plastic, have been borrowed to provide shelter; they have accepted invitations to work, they have collected money and have supported some people to make the route that consists in going to the center of the city.

Rooting and uprooting take place during all moments of violence and displacement, that is, their definition and temporal location is diffuse, given that many people or families have experienced these simultaneously, becoming a process of acceptance and adaptation that has entailed from having to leave something to adapting to a new world, which could be expressed in ceasing to exist in a world to inhabit or exist in a new one. The participating community leaders state that: *the mountain shelters us,* they want to construct *their mountain* or *the hillside speaks to the city,* but they do not only refer to the physical; they talk to *the hillside,* and testimonies affirm that this *is the best air you breathe in the city is here in the mountain, the most beautiful balcony; up here, we have no contamination*; all these adjectives are meanings they have given to their habitat. Under this perspective, those residing in the neighborhoods have already transcended from the dream of seeing it as they imagine it to building it with their own efforts, with their hands and those of their neighbors, with or without state interventions; they are aware that those ideals require time, making structural and behavioral transformations necessary. They have already traveled a path of sum of efforts: there are projects, leaders, networks, love, and identity. Reflections in the field diaries expose existence of other unhealthy processes that weaken the lives of groups and families, as occurs with phenomena, like landslides with land removal causing structural damage to sewers, access roads and homes.

### Singular dimension: families from the La Cruz and La Honda neighborhoods 

The singular dimension, from the voices of the families, revealed their needs, potentialities and, specially, the skills they have to move resources, the ways they have established support networks and the solidarity efforts they undertake day to day to survive with creativity, which has favored social and economic ties for adaptation and resilience. 

**Figure 1:**
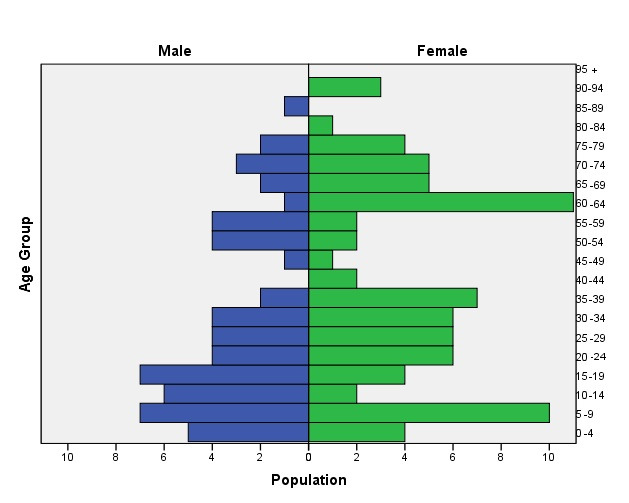
Population pyramid of 139 people from the La Cruz and La Honda neighborhoods. Medellín; 2020

The study had the participation of 40 families; 29 have suffered situations of displacement, some from diverse regions of the country and from the department of Antioquia, five with internal displacement in Medellín and three of them immigrants from Venezuela. These families are made up of 139 members, finding an average of four people per family, with ages ranging between 5 months and 92 years; the femininity index is 15 women per every 10 men. The population pyramid represented, in the families visited, a greater number of people < 40 years of age and a lower number in their 40s and 60s, a situation that can be explained by the processes of violence lived between 1980 and 1990, which meant disappearances, deaths and fleeing from the territory; people 60 years of age or older are mostly women, who - according to the information - settled there after being displaced with their children and in absence of their life companions (Figure 1). 

The dependency index showed that for every 100 people in productive age there are 81 people dependent on care; also, the aging index indicated that for every 100 people ≤ 15 years of age, there are 90 people > 60 years of age, which permitted unveiling intergenerational dynamics and the necessities for care that could be required in the mid to long term in this population. The marital status with the greatest representativeness was couples in free union and married, with 45%, followed by those who are single with 36%. The study obtained the educational level of 121 participants, finding that 20 of them are illiterate, 40 have basic primary studies, 55 secondary education, and six have higher education, a critical situation for human needs, like understanding, protection, and community and social participation.

The work found 92 people in economically active age, 28 of them with unpaid family work, 24 with informal labor, and 16 unemployed (Table 1); another 47 are in situation of economic dependence, who are students, caregivers of children and the elderly. Twenty-six people were found with labor relations without social security, corresponding to those working in informality as domestic workers, garment workers, construction workers, informal vendors and community workers. It was observed, in the neighborhoods, offers of services, like child care, laundry services, motorcycle and bicycle rental, hairdressing businesses, miscellaneous shops, stores selling food, and hardware stores; other families live off activities, like recycling and the route consisting in going to the city center where the get some food; in the market place the merchants donate what is going to perish and is useless for the following day.

**Table 1:** Type of occupation of 92 people from the La Cruz and La Honda neighborhoods. Medellín; 2020

Occupation	Frequency (%)
Unpaid family work	28 (30.4)
Independent informal work	24 (26.1)
Dependent formal work	17 (18.4)
Unemployed	16 (17.4)
Independent formal work	3 (3.3)
Community work	2 (2.2)
No data	2 (2.2)

It was found that for every 10 women there are six men unemployed and unpaid family work showed 27 women for every man. The registries of the field diaries indicate that the women from the territory are mostly mothers who are heads of household, with high workloads, greater problems of intrafamily violence and exclusion from opportunities to study and work. People value the work that becomes their fundamental basic need; nonetheless, the circle of inequity, scant educational offers, and lack of a decent work aggravate their situation of shortcomings, like food, education, public utility services, recreation, leisure, and enjoyment of playtime activities, which prevents a more harmonious development.

The protective processes of the families were represented most frequently by family participation in decision making (92%), knowledge regarding health care (79.5%), schooling for children and adolescents (79.2%), and care of housing, like cleaning, physical repairs, improvement of gardens, orchards, floors, roofs, and lighting (79.1%). The unhealthy processes found most corresponded to the barrier with the transportation service (61.4%), children in labor activities (54.5%), and difficulty in exercising the right to health and procurement of food (47.7%).

## Discussion

This research, from the perspective of the social determination of health, found forced displacement as a phenomenon that cuts across the social reality of people, families and groups in their interaction with the territory, from the general dimension to the singular, and which has affected the Colombian population in all regions and, specifically, in Antioquia. Conditions, like the armed conflict, dispossession of land and deterioration of life have been unhealthy processes that reproduce historically inequality, exclusion from means of production and necessary consumption for survival. An example of the foregoing are the families participating in this research who went from owning their lands to being dispossessed and without a roof over their heads, due to the heartbreaking phenomenon of violence and the abuse of power by armed groups, which in the words by Breilh,^3^ affect differentially people, families, and collectives within a complex context. 

Dispossession of land due to violence and displacement in Colombia has been addressed by Paredes^19^ who states that it is a phenomenon of class contradictions and economic interests that thrive in spaces and times and which deteriorate health. As in our study, the author expresses that farming peasant displacement is linked to territory struggles by armed groups and to the use of the land for production logics and that, therefore, the populations present extreme vulnerability that moves them to uprooting, a consequence of the geopolitical dispute, alienating them from their conventional modes of production. In Colombia, authors, like Molano A^20^ and Uribe MT^21^, state that violence is a structural phenomenon determined by political and economic causes; also, Ronderos MT^22^ exposes that violence is historical and has been transformed over time with the participation of different players, like common crime, narcotics trade, guerrilla groups, State forces, para-military groups, among others*;* and from the perspective by Breilh,^4^ violence generates dispossession with a practice of extraction of natural resources, under the accumulation model that produces extreme impoverishment, destruction of living conditions, and deterioration of the environment.

Similar to that found in our study, López, *et al.*,^23^ affirm that the general dimension subsumes the ways of lives of families and communities, in the particular and singular levels, translated into difficulties in access to education, health, dignified housing, and social inclusion, as well as the enjoyment of decent work and family and community well-being, which expresses social asymmetry in class, gender, and ethnic relations in rural and urban areas. 

This research revealed that the settlement took place as a result of fleeing and meant a transformation in ways of life, with constructions from resistance and resilience, a finding also described by Louidor^24^ when exposing processes of uprooting and rooting as expressions of ambivalent social realities, spaces of cultural heterogeneities, resulting from historical inequality and structural detachment. Other authors, like Borde and Hernández,^25^ in their research on social determination in Latin American cities, expose the existence of the denaturalization of the territorial order with long-term configurations, where the situations and relationships of the dynamics of human production and reproduction are tied to social constitutions with biased privileges and cyclical problems, mediated by power relations; nevertheless, according to the authors, these articulations generate life-promoting processes, of resistance and re-existence.

Another finding, herein, regarding the singular dimension, revealed that the working modes are also determined by power, access to education and to opportunities for human development; similar to that found by Fuentes, *et al*.,^26^ in which they explain marked spatial differences in manual workers and low-status routine occupations, all conditioned by geographic inequalities that differentiate access to education and income, a phenomenon contrary to that expected, but which is increasingly accentuated and consolidated.

The research presents the historical-social dynamics and their comprehension in space and setting, which make sociological and geographic sense, with transit from uprooting to new rooting, issues that in Colombia - within the framework of conflict and violence - have been described by Salazar *et al.*,^27^ Molano,^20^ Uribe MT^21^ and Ronderos.^22^ Although the people and families participating in this study long for their places of origin, this space they now inhabit gives them hope, progress, tranquility; where the construction of ties and networks, physical and of coexistence, make them feel useful. We could state that, according with Tuan,^28^ people have a “sense of place” where they carry out actions and decisions anchored to a behavior, in the individual, the collective, and in cultural perspectives, which transforms them and neighbors. 

As expressed by the leaders and participants in this study, during moments of adversity, human suffering, and dispossession “community living saves you”; actions like the invitation to collective work, collective construction, and solidarity are healthy processes that perpetuate the lives and dignity of the people, families, and human collectives. According to such and as expressed by Soliz:^29^ “territories are living socioecological settings that are transformed and - in turn - also transform the people”.

## Conclusion

The social determination of the health of families comprises the interrelation of historical-social and personal processes centered on the being that have unveiled in the general, particular, and singular dimensions. Their health has been determined by processes of violence, displacement, fleeing and by historical exclusion from modes of production and the social vulnerability they have endured, in turn, leading to few opportunities for their life projects and which place the families in conditions of inequity and inequality in the city they inhabit and that are not alien to the situation of the department of Antioquia, the country and the world during the last decades. The families have experienced a process of transformation of the territory with resistance, solidarity, rooting and collective constructions; however, there is an urgent requirement for political and social will committed to the inclusion of vulnerable people and territories in the construction and implementation of policies that forge well-being and flourishing development, as well as continuity of the territorial autonomy.
